# 

**Published:** 1898-05-07

**Authors:** 


**Notices of Births, Deaths, and Marriages** are inserted in this column at a charge of 2s. 6d. for an announcement not exceeding four lines.

### NOTICE TO CORRESPONDENTS.

All MS., Letters, Books for Review, and other matters intended for the Editor should be addressed The Editor, "The Hospital," The Hospital Building, 28 & 29, Southampton Street, Strand, W.C.

The Editor cannot undertake to return rejected MS., even when accompanied by stamped directed envelope.

### Notice to Advertisers and Subscribers.

All Advertisements, Orders for Copies of The Hospital, and Business Communications should be addressed to **THE MANAGER, (Not to The Editor),** The Hospital, The Hospital Building, 28 & 29, Southampton Street, Strand, London, W.C.

The Cost of Subscription, post free, is as follows:—Per week, 2½d. per quarter, 2s. 8d.; per half-year, 5s. 3d.; per annum, 10s. 6d. Foreign:—Per annum, 15s. 2d.; payable, in all cases, in advance.

**Notice.—Vol. XXIII. of "The Hospital" (October, 1897, to March, 1898), in handsome cloth binding, is now on sale at the Office of this Journal, price 7s. 6d. Cases for binding the Numbers contained in this Volume, 1s. 6d. Index to Vol. XXIII., 2½d., post free.**

**The Monthly Part for May is now ready. Price 6d., by post 8d.**

### SCRAPS AND GLEANINGS.

AT the Samaritan Hospital, Belfast, 181 in and 731 out-patients were treated.

DURING 1897 the income of the Lincoln County Hospital exceeded the expenditure by £208.

AT the Gorleston Cottage Hospital the income in 1897 fell short of the expenditure by some £115.

TWENTY-FIVE more patients were admitted in 1897 than in 1896 at the Abergavenny Cottage Hospital.

DURING the year 1897, 916 patients were treated at the Worcester Ophthalmic Hospital, viz., 56 in and 860 out-patients.

THE in-patients admitted to the Luton (Bute), Hospital in 1897 numbered 169. These, with the 18 brought forward from the previous year, made a total of 187 patients under treatment.

THE total number of members of the Salisbury and South Wilts Provident Dispensary for the year 1897 was 8,349, an increase of 75 on the previous year. 19,002 prescriptions were dispensed during the year.

THE Norfolk and Norwich Eye Infirmary had in its wards during 1897 137 patients. In addition 678 out-patients were treated. Altogether the patients numbered 100 more than they did in the previous year.

THE report of the Edinburgh Eye, Ear, and Throat Infirmary states that during the past year the alterations and improvements commenced in 1895, and which include the installation of electric light and refurnishing and extending the wards, have been completed.

No less than 1,415 in-patients and 22,059 out-patients wers treated at the Manchester Royal Eye Hospital during 1897. The continued increase in the number of patients has necessitated considerable structural alterations at the Oxford Street Hospital, and they are now approaching completion.

THE additional facilities for treatment afforded by the appointment of two assistant anæsthetists and the consequent extension of this branch of the work of the Victoria Dental Hospital, Manchester, from three days to six days a week has resulted in an increase in anæsthetic operations from 2,117 to 2,988 during 1897.

THE past year has been a busy one in Belfast so far as hospitals are concerned. First and foremost, the Victoria Hospital has been planned and £100,000 raised to erect it, the new Mater Infirmorum Hospital has been erected on the Crumber-road, and additions to the Eye and Ear and the Samaritan Hospitals have been opened.

A SUPPORTER of the Portsmouth Eye and Ear Infirmary towards the end of the year asked what the deficit on the year's working was. He was told it was £64. He immediately sent the treasurer a cheque for that amount, and so at once wiped out the 1897 deficit. If all hospital supporters did this the hospitals might soon be freed from debt.

THE wards at the Western Branch of the Brighton, Hove, and Preston Dispensary, according to the last report of that institution, still remain a heavy drain on the funds of the institution, but they are so greatly needed and so much appreciated that it is impossible to grudge the expenditure. It is, however, hoped that the wealthy residents of Hove will in future support the institution to a greater extent than they have done in the past.

THE total number of patients treated in 1896-7 at the Edinburgh Royal Infirmary was 9,628, as against 9,478 in 1895-6, while the average daily number of patients was 703. The number of outdoor patients treated in the waiting-rooms was 28,697, as against 28,110 in the year preceding. The ordinary receipts for the year came to £30,620, being practically the same amount as for the preceding year, and the expenditure for the year was £41,085, compared with £39,282 in the previous year, showing an increase of £1,803.

THE new pavilion for the treatment of women's diseases, to be erected at the Edinburgh Royal Infirmary, will accommodate about 80 patients, and arrangements have been made for setting apart 50 of the beds for the treatment of diseases peculiar to women. The cost is estimated at £39,000. The managers of the infirmary have also in contemplation—although they are not prepared to go on with the work at present—the erection of two other pavilions. One of these is to be devoted to the treatment of ophthalmic complaints, and the other is to a large extent to be for skin diseases.

JUST PUBLISHED, with 394 Illustrations, Large Crown 8vo., 18s.

### A HANDBOOK OF MIDWIFERY.

**By WILLIAM RADFORD DAKIN, M.D., F.R.C.P.,**

Obstetric Physician and Lecturer on Midwifery at St. George's Hospital, &c.

"The most important addition for some years to the list of educational works on midwifery. . . . The author has succeeded in his aim of producing what is eminently a practical book, while for the purposes of the student with examinations in view it may be safely recommended as a reliable and sufficiently comprehensive text-book."—*Edinburgh Medical Journal.*

LONDON: LONGMANS, GREEN, AND CO.

